# Supplementary material for: Are carbon emissions trading and green financial instruments synergistic? -Comprehensive quantitative research based on content analysis
Source: PLoS One. 2024 Mar 7;19(3):e0298601. doi: 10.1371/journal.pone.0298601 (PMC10919601; doi:10.1371/journal.pone.0298601)
Supplement: S1 Data — (ZIP) [file pone.0298601.s001.zip › Data/地级市Z_F工作报告.zip!/─┌├╔╣┼/║⌠║═║╞╠╪╩╨/└┤╫╘╣1⁄2╓┌║┼í╛┬φ┐╦╩2╛▌═°í┐_2017.docx]

各位代表：

现在，我代表市人民政府向大会报告工作，请予审议，并请各位政协委员和列席会议的同志们提出意见。

一、2016年工作回顾

2016年是“十三五”开局之年。在自治区党委、政府和市委的坚强领导下，我们全面贯彻党的十八大和十八届三中、四中、五中、六中全会精神，认真学习贯彻习近平总书记系列重要讲话精神和考察内蒙古重要讲话精神，全面贯彻落实自治区党委、政府和市委的决策部署，主动适应经济发展新常态，践行发展新理念，深度推进供给侧结构性改革，实施“四个服务、两个保障”，统筹推进稳增长、促改革、调结构、惠民生、防风险各项工作，较好地完成了全年目标任务，实现 “十三五”良好开局。

——经济发展稳中有进、走在前列。全市地区生产总值完成3173.6亿元，增长7.7%；规模以上工业增加值增长9.1%；固定资产投资完成1849.2亿元，增长14.2%；一般公共预算收入完成269.7亿元，增长9%；社会消费品零售总额完成1481.5亿元，增长9.5%。地区生产总值、规模以上工业增加值、一般公共预算收入增速均居全区第3位，保持中高速增长。

——新兴产业发展迅速、结构优化。新增规模以上工业企业23户，规模以上工业企业实现利税和利润同比分别增长20.7%和54%；工业税收占比达到45%。新增高新技术企业43家，总数达到131家，战略性新兴产业占规模以上工业产值比重达到30.4%；三次产业比重调整为3.6∶27.8∶68.6，经济发展质量和效益明显提升。

——城乡建设统筹推进、亮点纷呈。全年完成城市建设投资691亿元，同比增长69.8%。完成征拆面积847万平方米。全面建成65公里城市快速路，地铁、高铁、机场迁建等一批重大工程加快推进。总投资130亿元，完成1400个村的基础设施和公共服务建设。启动和林格尔新区建设，治山、治水、治气等环境治理取得阶段性成果。

——人民生活持续改善、稳步提高。全市各项民生支出达到293.9亿元，占财政总支出的70%；城镇常住居民人均可支配收入40220元，增长7.7%；农村常住居民人均可支配收入14517元，增长7.6%；城镇新增就业4.1万人，16909名农村贫困人口实现动态稳定脱贫，全面建成小康社会取得显著成效。

一年来，我们主要做了以下工作：

（一）深度推进供给侧结构性改革。下大力淘汰落后产能、改造传统产能。拆除70万吨落后水泥生产线，依法关闭26户“小散企业”，引导阜丰生物、齐鲁制药等企业实施产品升级技术改造，推动生物医药产业整体转型升级。多措并举消化房地产库存。建立了房地产购销平台和房屋租赁平台，将棚改货币化安置率提高到90%以上，放宽住房公积金使用条件，积极培育房屋租赁市场，大力发展创客地产，租赁改造33万平方米商业办公用房建设创业园、产业园。全年商品房成交面积达到559.9万平方米，同比增长37.5%。综合施策降低企业运行成本。在简政放权、减税降费、电价市场化改革、创新融资方式、降低物流成本等11个方面打出“组合拳”。认真落实国家电价补贴政策，将17户企业纳入自治区电力多边交易平台，两项合计为企业降低用电成本3.6亿元。积极帮助困难企业开辟融资渠道、开拓市场，为40户中小微企业发放“助保金贷款”2.3亿元，帮助31户停产半停产企业实现复产。高度关注金融风险。下大力化解政府债务，争取自治区置换债券资金286.8亿元，减少债务利息6.9亿元，一般债务率下降至75.9%。加快补齐发展短板。实施了一批打基础、利长远的重大项目，全年亿元以上项目开复工322个，累计完成投资843.9亿元。

（二）大力培育发展新经济新动能。按照“科技+基金+大数据+文化”的发展模式，着力培育发展以乳业为主导的绿色食品产业，以光伏材料为龙头的新材料产业，以草、树为特色的生态环保产业，以新能源汽车为代表的先进装备制造产业和以云计算为基础的大数据应用产业。建成乳业、蒙草种业科技、矿业科技等7家研究院，正在规划建设晶体研究院，加快推进中环光伏四期、沃特玛新能源汽车产业创新联盟、环聚新能源等一批项目，引入了航天云网、百度、华为等大数据应用项目，全市云计算服务器装机能力达到70万台，新能源、新材料等战略性新兴产业实现产值440.9亿元。加快发展现代服务业。培育了中国薯网、华蒙通等20多个大型交易平台，电子商务交易额790亿元，网络零售额175亿元，分别增长49%和28.7%。新增各类金融机构8家，金融机构存贷款余额分别增长15.2%和16.1%。举办各类展会98场，成交及意向协议总额超过135亿元。完成城发恼包快递电商产业园一期、4个旗县快递集散中心和60个末端配送网点建设，“天津无水港”成功落户沙良物流园区，全市公路货运量和货运周转量分别增长9.4%、8.3%。开工建设14个文化重点产业项目。全年接待旅游人数和实现旅游收入分别增长13%、21%。着力发展现代都市农业。本地蔬菜供应率达到67.8%，同比提升10个百分点。农牧业产业化龙头企业达到324家，全年销售收入1560亿元，规模和效益均居全区首位。积极推动创新创业创意。设立了8亿元创新奖励扶持资金和10亿元创新投资引导基金，带动社会投入73亿元促进创新创业，全年新增自治区新型研发机构1家、第二批众创空间试点基地8个，新增新三板挂牌企业11家，成功申报国家小微企业创业创新示范基地城市，内蒙古大学生创业园被确定为全国创业孵化示范基地。全市专利申请量和授权量分别增长22.5%和12%，万人拥有有效发明专利5.4件，居全区首位。

（三）着力提升城乡建设管理水平。一是城乡规划体系不断健全完善，编制完成各类规划8项。二是城市综合立体交通体系加快形成。机场迁建项目获得国务院、中央军委批复，地铁1、2号线一期工程全面开工，完成4.7公里地下综合管廊建设，打通45条断头路。优先发展公共交通，以租代购增加新能源公交车427台，完成BRT快速公交站点建设，新开通公交线路7条，延伸调整线路19条，将公共交通机动化出行分担率提高到48%。三是城市功能进一步完善。加快推进少数民族群众文化体育运动中心、国家（北方）足球训练基地和儿童探索博物馆等一批功能性场馆建设。四是城市文化、民族特色进一步彰显。加快如意总部区块、将军衙署区块、昭君博物院区块改造建设，完成大南大北街等特色景观街改造。五是老城区通过拆改建旧貌换新颜。深入推进拆改建、交通管理三年行动计划，重点对快速路两侧、机场路及河道水系两侧脏乱区域进行征拆和绿化。完成284个老旧小区改造、75个片区老旧小区供排水管网及二次加压设施改造。六是城市管理水平进一步提升。集中开展城市环境卫生综合整治提升行动，落实网格化、精细化管理责任，对13条主次街道实施街景整治，打造31条示范小街巷、301个示范社区，实现中心城区209平方公里标准化管理全覆盖。七是城市生态环境明显改善。完成4座污水处理厂提标扩建工程和4段城市黑臭水体治理，城市污水处理率由91.8%提高到95%以上，再生水回用率由20%提高到35%以上，地表水国控考核断面水质达标率达到75%。全面启动城市水系生态建设工程，总投资43亿元的大黑河综合治理工程实现注水。集中全力治理大气污染，实施京能盛乐电厂热源入呼工程，淘汰改造燃煤锅炉222台，PM2.5年平均浓度同比下降4.7%，空气质量优良天数同比增加7天，达到283天。深入实施“三环两带”生态绿化工程，建成丝绸之路公园和9个山体公园，全年完成绿化面积43.2万亩，建成区绿化覆盖率达到38.3%。八是美丽乡村建设与脱贫攻坚工程取得阶段性成果。改造农村危房70365户，建设标准化卫生室310个、文化室420个，完成322个村的农村电网改造，新建改造水源590个、管网4499公里，解决24万人的安全饮水问题，完成街巷硬化5622公里，具备条件的行政村全部通上沥青水泥路。完成呼塔二期等公路建设，新增公路里程434公里。全力推进精准脱贫攻坚工程，新建易地搬迁和生态移民房屋6127套，发放金融扶贫贷款1.6亿元。

（四）全面深化改革开放。重点推进经济生态领域19大类211项改革任务，形成一批改革成果。深入开展武川县矿产资源资产责任审计，完成清水河县自然资源负债表森林、湿地、草原实物量登记工作。深化商事制度改革，率先在全区启动 “五证合一、一照一码”改革，全年新增各类市场主体5.1万户。持续深化国企改革。稳步推进农村综合改革，和林县农村土地征收制度改革试点在“非典型地区”试出了“典型经验”，在赛罕区开展了农村宅基地、土地承包经营权抵押贷款业务，全面完成武川县农村集体土地承包经营权确权登记颁证试点工作。深入推进户籍制度改革，实施居住证制度。全面推开不动产登记。积极有效推进文化体制改革。深入推进城市管理体制改革，有效整合城市管理和执法职能。按要求完成了行政单位和参照公务员管理事业单位公务用车改革。创新投融资体制改革，全市PPP项目投资总额达到1134亿元，有力保障了全市重点项目建设。深化财税体制改革，全面实行“营改增”。不断深化医疗卫生体制改革，公立医院药品、耗材、大型检查收入占总收入比重明显下降，仅取消药品加成一项让利患者近3亿元。进一步扩大对外开放。组团参加香港、广东等地举办的经贸合作活动，在北京成功举办大数据产业推介会，全年共引进项目330项，到位资金706.6亿元。大力发展跨境电子商务，开通首条国际快件俄蒙出口通道，进出口快件数量是2015年的4.4倍，增速在全国31个开通国际快件业务城市中排名第1位。

（五）持续保障和改善民生。积极促进就业创业。筹集就业专项资金2.9亿元，发放创业担保贷款1.6亿元，帮助2.1万名高校毕业生和5479名就业困难人员实现就业，转移农村劳动力就业27.4万人。完善社会保障体系。全面启动机关事业单位养老保险改革，为18.5万名企业退休人员调整养老金，人均月增资167.3元。实施大病补充保险制度，统一城乡居民医保个人缴费标准。全年城乡低保保障12.1万人，医疗救助4.5万人（次），教育救助1852人（次），临时救助1.8万人（次），参保参合救助12.7万人，发放救助金4亿多元。努力办好人民满意的教育。新建、续建23所公办中小学、幼儿园，4个县区顺利通过全国义务教育发展基本均衡县评估认定。面向社会公开招聘教师629名、农村特岗教师20名。校园网络覆盖率和多媒体教学设备覆盖率分别达到55%和87%。全面推进健康呼和浩特建设。推进实施11个卫生重点项目，市蒙中医院、妇幼保健院和新建的6个社区卫生服务中心投入使用。深入实施乡村医生巡诊制度，惠及偏远地区群众2.8万人。加快推进游泳跳水馆、体育馆及一批小型文体馆建设，人均体育场地面积达到2.2平方米，是全国平均水平的1.5倍，市区基本实现了“15分钟健身圈”。着力推动文化事业繁荣发展。完成54个乡镇（街道）综合文化站和703个村（社区）基层综合性文化服务中心达标建设，成功举办第六届中国·呼和浩特少数民族文化旅游艺术活动、第十七届昭君文化节，推出《昭君》《巡城记》等一批优秀剧目，民族文化的影响力和感召力进一步提升。

（六）努力维护社会和谐稳定。始终把安全稳定放在重要位置，全力推进平安首府建设。加强和创新社会治理，打造了30个创新服务示范社区，完成第九届社区居委会换届选举工作。推行信访首办责任制、领导包案、阳光信访等制度，着力解决回迁安置、拖欠农民工工资等突出问题，信访批次和人次分别下降10.8%和11.7%。加快实施“平安365工程”，基本完成平安首府视频监控联网应用项目建设，全市刑事案件发案下降11.1%，全年有239天无“两抢”案件发生。强化生产安全监管，全面开展安全生产大检查和重点领域、重点企业专项整治，全年安全生产事故起数下降28.4%。强化食品药品监管，完善食品药品监管网络体系。积极支持国防和军队现代化建设，注重军民融合式发展，国防动员、民兵应急力量建设得到加强。民族、宗教、人防、气象、防汛、防震减灾、外事侨务、工会、疾控、妇女儿童、计划生育、青少年、老龄、残疾人、关心下一代、红十字、慈善、史志、档案等各项工作取得了新成绩。

（七）加强高效法治廉洁政府建设。加大“放管服”改革力度，实施审批流程再造，审批时限平均减少29.4%，我市公共服务满意度在全国38个上榜城市排名第3位。严格执行“三重一大”集体决策制度，推行政府法律顾问制度，强化重大行政决策合法性审查，依法行政水平得到全面提升。推进社会主义民主政治建设，认真执行市人大及其常委会的决议决定，主动接受法律监督和工作监督，自觉接受市政协民主监督，积极支持市政协开展民主协商，认真办理人大代表建议、政协委员提案，办复率达到100%。进一步深化政务公开，定期通报全市经济社会发展情况，主动回应社会关切，全年举办各类新闻发布活动116场。严格落实从严治党主体责任和党风廉政建设各项规定，“三公”经费压减11%。加强行政监察和审计监督，完成对24个新农村建设项目和市本级政府投资项目的审计工作，核减投资额1.3亿元。深入开展“两学一做”学习教育，持续改进工作作风，政府班子的凝聚力和战斗力有了新的提高。

各位代表！过去一年的成绩，是在全国经济下行压力增大、周边地区经济深度调整、全区经济进入快速增长消化期的形势下取得的，是自治区党委、政府和市委正确领导的结果，是全市各族人民齐心协力、团结奋斗的结果，也是社会各界大力支持、大力帮助的结果。在此，我代表市人民政府，向辛勤工作的全市各族干部群众，向人大代表、政协委员，各民主党派、人民团体，向所有关心支持首府建设与发展的同志们、朋友们，表示崇高的敬意和衷心的感谢！

在肯定成绩的同时，也清醒地看到面临不少困难和问题。一是经济下行压力依然较大，工业短板仍未补齐，新经济新动能尚未完全形成，稳增长、调结构的任务还很艰巨。二是城市综合承载力和竞争力还不够强，市政基础设施欠账多，城市管理不够精细，交通拥堵等“城市病”还未很好地解决，城市规划建设管理水平有待进一步提高。三是生态建设和环境保护压力较大，水、大气、土壤等污染治理任务依然艰巨，推动绿色发展还需持续发力。四是城乡居民收入还不高，农民持续增收面临较大压力，部分群众生活还比较困难，脱贫攻坚、移民搬迁、农村产业发展需下更大气力。五是少数领导干部存在为官不为、不敢担当的现象，政府自身建设还需进一步加强。对于这些困难和问题，我们要以创新的思维、必胜的勇气，下大力气解决。

二、2017年工作任务

当前，我市正处于机遇叠加、蓄势奋发的重要时期，国家“一带一路”倡议向纵深推进，自治区成立70周年大庆助推首府各项建设，以大数据为代表的新产业新经济时代已到来，以国家级新区建设为代表的新动能新机遇已到来，以地铁、机场、高铁、高速公路、快速路网为标志的快捷、立体、综合交通时代已到来，全智慧、全过程、全员化的新城市管理时代已到来，首府将迎来一个前所未有的崭新发展阶段。

今年政府工作的总体要求是：全面贯彻党的十八大和十八届三中、四中、五中、六中全会精神，深入贯彻习近平总书记系列重要讲话精神和治国理政新理念新思想新战略，认真落实自治区第十次党代会和市第十二次党代会、十二届二次全会决策部署，统筹推进“五位一体”总体布局、协调推进“四个全面”战略布局，坚持稳中求进工作总基调，适应把握引领经济发展新常态，以提高发展质量和效益为中心，以推进供给侧结构性改革为主线，瞄准“一个总目标”、守住“三条底线”、践行“五大理念”、促进“五化协同”、抢抓“五大机遇”、构建“五大体系”、打好“五场硬仗”、突出“五种导向”，谋在新处、干在实处、走在前列，提升首府“首位度”，努力打造区域发展重要增长极、现代化区域性中心城市、国家向北开放桥头堡的重要支撑、和谐宜居幸福的示范区与更具活力、更为美丽、更加和谐的首善之区，以优异成绩迎接党的十九大胜利召开，庆祝自治区成立70周年！

今年发展的主要预期目标是：地区生产总值增长7.5%左右，规模以上工业增加值增长9%左右，固定资产投资增长16%左右，一般公共预算收入按可比口径增长6%左右，社会消费品零售总额增长10%左右，城乡常住居民人均可支配收入分别增长8%和8.5%左右，全面完成自治区下达的各项工作任务。

实现上述目标，重点要做好以下工作：

（一）全力推动和林格尔国家级新区建设，加快打造发展新引擎。牢固树立强烈的发展意识、机遇意识，举全市之力、全区之力，借全国之势、全国之智，推动国家级新区建设，为首府和全区发展配置动力强劲的世界级发动机，与发达地区站齐、看齐，争取以自身的“一马当先”“一往无前”，带动自治区发展的“万马奔腾”“万象更新”，实现走在前列、打造祖国北疆亮丽风景线的目标。

科学谋划新区发展。按照打造我国向北开放桥头堡的重要支撑、创新体制机制的重要平台、辐射带动区域发展的重要增长极、产城融合发展的重要示范区、绿色循环低碳发展的生态智慧区、民族地区繁荣发展的先行区等发展定位，将6个旗县区496平方公里纳入新区建设范围。统筹整合新机场组团、盛乐组团、沙尔沁组团，培育产业集群，完善城市功能，打造新区发展核心区；协同推进金桥组团、白塔组团产城融合发展进程，加快完善托清组团工业基础设施配套，南北两翼双向发力，全面拉开新区建设框架，全力构建“一核两翼六组团”新区发展格局。

按照“一年起步、三年成型、五年上水平、十年大跨越”的发展目标，以“开局就是决战、起步就是冲刺”的劲头，通过全球招标高起点、高水平完成新区总体规划和各类专业产业规划编制，构建生态化的规划体系、市场化的管理体系、国际化的政策体系。组建投融资、产业发展和基础设施建设三大公司平台，争取自治区尽快研究出台加快推进新区建设的实施意见及分工方案，并分别设立200亿元新区发展和建设基金。坚持先规划后建设、先地下后地上、先生态后业态、先生产后生活，全面推进新区基础设施和公共服务配套设施建设。加快“一山两库五河”治理，推进综合交通枢纽、高铁、高速、高架路网等“七网”建设。开工建设内蒙古艺术学院、大数据学院、职业教育学院及实训基地项目，同步推进国际化教育、医疗等公共服务设施布局。

以新区建设为依托，积极申报建设陆路国际港务区、中国—蒙古国博览会永久会址、内陆港铁路口岸、综合保税区、海关监管区、临空经济区、自由贸易区等对外开放平台，开展国际贸易结算中心试点、期货保税交割试点工作，开工建设大匠园区、大数据集中区、网货生产园区、民政福利产业园区等产业、科技聚集区，引进建设一批高端产业项目，助推新区发展全面起步。

（二）全力培育新兴产业，加快构建具有首府特色的现代产业体系。紧盯新一轮科技革命和产业变革方向，多维度促进新兴产业和主导产业发展，全方位推动三次产业融合，激发新动能，培育新支撑，促进产业发展迈向高端化、智能化、绿色化、服务化。

大力发展战略性新兴产业。积极申报“中国制造2025”试点示范城市，推动高端装备制造、大数据、新材料、新能源、生物医药等新兴产业发展壮大，全年完成产值470亿元以上，持续引领产业迈向中高端。围绕新能源汽车生产基地建设，确保沃特玛新能源汽车产业园一期项目全面建成投用，开工建设蓝海华腾电机控制器、10万吨三元锂电池制造等18个二期项目，建成具有明显竞争优势的动力电池产业链和电动车整车制造全产业链，促进高端装备制造产业快速发展。实施大数据发展工程，组建大数据发展管理局，成立大数据发展公司，服务器装机能力达到100万台，推进产业数字化、城市建管智能化，全力打造“中国云谷”。发挥光伏材料集聚形成的成本优势、规模优势、技术优势，完成晶体研究院、国家检测中心建设，促进全产业链高端化。围绕航天新材料、碳纤维复合材料等领域开展协同应用试点示范，加快石墨烯、富勒烯碳纳米等产业基地建设，推动形成“碳、硅、铝、锂、钴”等为主的新材料产业群。深入推进清洁能源生产应用，启动环聚二期、联合新能源等项目建设，持续推进光伏扶贫工程建设，着力打造清水河、武川光伏产业应用基地，新能源产业占工业总产值比重超过20%。加快生物产业创新发展步伐，壮大蒙中医药、动物疫苗、生命干细胞等产业规模，打造一流的生物医药研发生产基地。

加快构建服务业“四大中心”。继续做好域外金融机构引进工作，推动发展第三方支付、众筹众扶等互联网金融产品，扶持建设金融资产管理、融资租赁等新型金融主体。大力发展总部经济，优化政策措施，引入更多企业总部落户我市，建设西北地区重要的金融交易结算中心。改善提升“两港三区”基础配套，加快快递电商产业园、大宗物资仓储基地、零担企业集聚区建设，打造物流公共信息平台，培育引进一批大型物流企业，新增1000个城乡物流配送终端网点，建设服务呼包鄂、连通京津冀、面向俄蒙欧的现代仓储物流配送中心。大力培育品牌展会，办好第二届中国—蒙古国博览会、第十届中国民族商品交易会等大型展会，筹办国际乳业博览会等一批新型展会，开工建设呼和浩特会展中心，展会场次和交易规模分别达到120场和200亿元。实施“旅游+”战略，全面统筹旅游资源开发建设，编制“全域全时旅游”发展规划，开展全域旅游示范旗县区创建活动，昭君博物院改造等项目投入运营，八拜湖旅游景区、蒙元世界草原文化产业园和冬季旅游项目建设取得重大进展，营造处处是风景、四季好风光的旅游全新格局。稳步推进养老服务市场化改革，积极推进政府购买居家养老服务，加快与养老有关的智慧社区、医养结合基地建设，打造北方地区重要的会展文旅健康中心。完善创业创新政策体系，落实《大众创业万众创新实施意见》，围绕战略性新兴产业发展新建一批 “双创”示范基地和专业化文化创意众创空间，依托互联网打造一批开放共享的跨界协同创新平台，各类市场主体达到22.8万户，高新技术企业达到140家，建设领先全区、引领周边、面向全国的创新创业创意中心。

不断激发特色主导产业新活力。继续支持乳业、草业、肉业等绿色产业企业充分利用国内国外两个市场，做大总部基地生产能力，提升产品研发和竞争能力，增强原料市场掌控权、消费市场主导权、国际研发市场话语权，巩固提升“中国乳都、草都、肉羊第一品牌”核心地位。推动大中型热电联产、风电、光伏发电等能源产业规模化、集约化发展，支持电力生产企业开展市场供需对接，提高生产荷载和效率，打造全国重要的清洁能源输出基地。

（三）积极扩大有效需求，全力保持经济平稳增长。精准加力有效投资，不断激发市场活力，促进经济稳中有进、进中向好，坚决守住发展底线。

持续释放和扩大投资拉动力。牢固树立“项目第一”的发展思路，组织开展春季、秋季重大项目集中开工活动，全年实施亿元以上重大项目468项，力争完成投资1481亿元。积极优化投资结构，加大工业投资强度，提高战略性新兴产业投资比重，提升民间投资积极性，以投促快、以投调优，工业固定资产投资、战略性新兴产业投资和民间投资增幅均高于全社会固定资产投资水平，全年新增规模以上工业企业15户。

着力拓宽投融资渠道。创新项目建设投融资方式，完善政府和社会资本合作机制，加快推进产业融资PPP模式，整合组建总规模为100亿元的产业引导和风险投资基金，放大财政资金的杠杆效应，突出社会资金的增值效应。规范国有融资平台运行，厘清功能定位、突出主业发展、优化债务结构。发展多层次资本市场，开展上市企业、科技小巨人企业培育行动，支持企业在主板、新三板、区域性股权交易市场挂牌，鼓励企业通过发行债券和票据进行融资，市场直接融资规模力争超过300亿元。

不断挖掘消费潜力。顺应消费需求变化新趋势，继续增加文化信息、旅游休闲、体育健身等服务项目，积极开拓医养结合、健康管理、康复护理等服务模式，推动服务业模式创新、跨业融合、外延拓展，促进服务多元化供给与多样化需求更好对接，着力扩大服务消费。以工业消费和食品为重点，推动全面质量管理，加强企业的认证认可和标准化建设，增加高质量、高水平的有效供给，扩大中高端产品消费，促进消费扩大升级。

（四）发力农业供给侧，加快农业现代化进程。持续推进创新强农、绿色兴农、协调惠农、共享富农，大力推动农业供给侧结构性改革，让农业有奔头、农村有看头、农民有干头。

强化绿色供给满足绿色需求。加快推进绿色农畜产品生产基地建设，培育特色产业集群，完善批发交易市场体系，打造西北地区绿色农畜产品集散地。实施奶牛牧场标准化建设和管理“双标”提质工程，奶牛养殖全部实现规模化，年单产提高1吨，40%以上牧场实现电子化管理。启动建设全区肉羊交易市场，鼓励发展肉羊工厂化屠宰加工，规模化养羊比例达到60%，加快肉羊产业化发展进程。抓好首府 “菜篮子”工程，本地蔬菜供应率稳定在70%左右。合作设立100亿元草业发展基金，支持开展生物育种、草种资源库建设，鼓励企业加大营养型、耐寒型、节水型牧草品种培育种植力度，及时向生态绿化、体育用草等专业领域进军，推动草产业向“种养”一体、“种加”一体、“种赏”一体发展。积极开发绿色生态休闲产品，加强森林、湖泊、草原、湿地的修复保护，加快农家乐、牧家乐建设，大力发展观光型、融合型、智慧型、旅游型、生态型农业，年内新建9处综合性农业观光区。开展小香米、土豆等特色农产品标准化生产示范，建设一批原产地保护基地，培育一批知名合作社品牌和农户品牌，推动现代农业全产业链增值和品牌化发展。

加快转变农业发展方式。加大实用人才带头人、现代青年农场主、农村青年创业致富“领头雁”培训力度，实施农民工等人员返乡创业行动计划。年内培养农民专业合作社200家、家庭农场200个、新型职业农民1000人。加快农业产业化进程，推动农民合作社、家庭农场与龙头企业、配套服务组织集群集聚，推动土地经营权入股发展农业产业化经营，产业化龙头企业年销售收入突破1650亿元。加快推进电子商务进农村，引导新型经营主体对接各类电商平台，农产品网上零售额占农业总产值比重达到6.2%。健全完善农牧业科技服务体系，加快农牧业科技成果推广应用。实施化肥农药使用量零增长行动，推进农业废弃物资源化利用，走产出高效、产品安全、资源节约、环境友好的农业现代化发展之路。

大力推进脱贫攻坚。坚持精准扶贫、精准脱贫基本方略，强化问题导向，聚焦薄弱环节，深入落实“六个精准”要求和“五个一批”举措，加快完善贫困户动态管理制度，瞄准贫困问题最突出的区域、群体和环节，因地、因人、因事施策。支持重点贫困地区统筹整合各级财政安排的农业生产发展、农村基础设施建设资金，允许特殊贫困人口叠加享受各类惠民政策。加大产业、就业扶贫力度，提升精准兜底保障能力，培育壮大贫困村集体经济，多渠道促进贫困人口增收，建立健全稳定脱贫长效机制，确保今年全面完成移民搬迁任务，率先在全区实现建档立卡贫困人口动态脱贫，武川县和清水河县两个贫困县全部摘帽。

集中打好新农村建设硬仗。力争用三年时间，完成城中村、城边村农民就地城镇化工作，完成常住户数20户以下自然村搬迁转移工作。加快农村污水管网和小型生态处理设施建设，健全完善基础设施和公共服务投入、管护长效机制，巩固农村基础设施建设和环境建设成果，推进乡村生态化、园林化、绿色化。

（五）健全完善基础设施体系，不断夯实发展基础。按照 “七网”建设要求，持续加大基础设施建设投入力度，在全区率先实现“七网”全覆盖。

加快打造“大交通”发展格局。全面开工建设和林格尔新机场，加快推进老牛湾通用机场项目。以新机场为核心，启动市区-新机场-和林-清水河、清水河-呼准鄂既有线、武川-火车东客站、托县-新机场动车项目，加快筹建环城连县轨道交通，加快构建全市域动车路网。推动呼张高铁、呼准鄂快速铁路建成通车，加快推进呼市-银川、呼市-太原、呼市-南宁等重点高铁建设项目。开工建设市区-新机场、呼市-鄂尔多斯、新机场-清水河城关镇、新机场-察素齐镇高速公路，加快推进国道209线武川-呼市大青山隧道及引线工程、国道110线呼市-毕克齐-协力气一级公路、呼市-毛岱公路、沿黄公路托县-老牛湾段、省道311线武川-杨树坝一级公路等重点项目建设，着力构建“市内大循环、外围大辐射、区域大联通”的现代化立体综合交通网络，打造国家级综合交通枢纽中心城市。

加强市政、水利、能源和通信等基础设施网建设。完善城市地下管网规划，铺设给水管网100公里、雨水管网171公里、污水管网148公里、再生水管网17公里，开工建设新区地下综合管廊，进一步完善市政设施功能，提高基础设施承载水平。投资10亿元，实施大型灌区续建配套和节水改造、病险水库除险加固、水土保持治理等重点农区水利工程，启动引黄河水入新区工程，有效提升水资源保障能力。实施新一轮农网改造升级，农村电网供电可靠率达到99.8％，综合电压合格率达到97.3％。加快“智慧呼和浩特”建设，深入推进“宽带中国”示范城市创建，提高宽带覆盖水平、网络安全保障水平和信息资源综合开发利用水平。

（六）强化生态环境综合治理，筑牢生态安全屏障。牢固树立“绿水青山”就是“金山银山”的发展理念，以落实中央环保督查反馈意见整改为契机，持续加大生态保护建设力度，坚决守住生态底线。

突出治气。全面推进供热体制改革，积极推动“厂网分离”。完成京能盛乐热电一期、启动盛乐热电二期、大唐托电等热源长输入呼工程。拆并整合城区剩余79座分散燃煤锅炉房，推广使用天然气、地热等清洁能源。完成大唐托电、金桥电厂、科林电厂超低排放改造任务。强化汽车尾气污染治理，推广清洁能源汽车，新增电动公交车306辆，有效应对空气污染，提升空气质量。

继续治水。落实最严格的水资源管理制度，重点抓好引黄入呼三期工程，强制关闭城市公共供水管网覆盖范围内自备水井，全面推进分质分类供水，提高再生水利用水平。加快推进雨污分流、雨水截留、黑臭水体整治，继续实施“五河两湖”城市生态水系建设工程，推进河道综合整治和生态恢复，有效改善区域水环境面貌。加强污水处理设施建设，完成公主府污水处理厂深度处理及3座旗县污水处理厂扩建提标工程，城区污水处理标准全部达到一级A+，基本实现城区污水全收集全处理，城镇截污全覆盖。

重拳治山。加快实施“揽山入城”战略，开展全民绿化大行动，投资100亿元，全面推进大青山前坡400平方公里生态保护综合治理工程，大力发展休闲度假、健康养生、文化旅游等特色产业。全部关停大青山保护区内采矿、采沙、采石等企业，从根本上解决大青山私挖乱采等破坏生态行为。全面清理整顿清水河县采矿企业，推进采矿企业合法、规范、安全运营。

大力推进生态产业化、产业生态化。继续组织实施京津风沙源治理、天然林保护和新一轮退耕还林等重点生态工程，年内完成营造林面积40万亩。大力推进城市湿地、城市园林、城市绿地、城市绿道等建设，充分利用闲置土地、荒山坡地开展植树造林，全面提升“三环两带”、出城口等重点区域绿化水平，年内新增公园、游园10个，新增城市绿地5000亩。启动清水河百万亩经济林种植行动计划，使之成为生态林、脱贫林、小康林。坚持绿色发展，用绿色化理念改造提升现有产业，进一步做大做强草、树等生态产业，引导推广绿色低碳环保的生产生活方式，推动形成产业生态化与生态产业化互动互促的发展新格局。

（七）强化城市建设管理，不断提高首府宜居水平。以争取联合国人居环境奖为目标，持续加大城市建设改造力度，打造便捷整洁、宜居宜业的现代化首府城市。

全力推进主城区改造提升。继续推进“拆改建三年行动计划”，投资323亿元，完成678.8万平方米棚户区改造任务；公开摇号分配公租房4200套；完成1.3万套回迁房续建任务；对158个老旧小区实施改造提升工程，拆出来的区域全部建成绿地公园和功能设施；加快推进老旧小区物业管理全覆盖，对300个准物业管理老旧小区实施市场化、专业化物业管理。高标准规划建设3000个左右公共厕所，提高便利服务功能，让首府群众生活更舒适、更安心。

铁腕治理交通拥堵。启动建设中心城区“十字形”快速路和云中路新机场连接线、外三环等城市快速路，完成滨河环路准快速化改造，完成大庆主会场连接线、白塔机场连接线项目，进一步完善城市快速路网体系。加快推进地铁1、2号线建设，启动实施地铁3、4号线项目，努力让首府地铁时代早日到来。优先发展城市公交，新建火车东客站公交综合场站等6座公交场站，优化调整公交线路，实现中心城区步行5分钟到达公交站点。在人员密集、交通流量大、集中拥堵区域，新建续建公铁立交桥7座、跨河桥4座、过街天桥20座，有效缓解交通拥堵。针对不同区域、不同路段，研究制定区划分流规划，进一步提升城市道路通行能力，让首府市民出行更顺畅、更便捷。

强化城市面貌综合整治。以新华大街、机场路、成吉思汗大街等重要街区综合整治为重点，改造提升22条主次干道、258条小街巷，美化亮化13座公铁立交桥。整治规范餐厨垃圾等收集、清运，做到无害化处置，建成各类环卫作业场站及生活垃圾中转站5座，建成生活垃圾处理场4座，基本建立餐厨废弃物、建筑废弃物回收和再生利用体系。坚定不移开展城市环境综合整治专项行动，加强长效化、常态化管理，让首府环境更整洁、更优美。

加强小城镇建设。以五个旗县政府所在地城镇为重点，围绕各自不同特点和优势，加大基础设施建设投入力度，有效提升公共服务水平，大力发展县域经济，提高城镇吸纳辐射带动能力，努力打造一批人口集中、产业集聚，特色鲜明、充满活力的特色化、专业化小城镇。

（八）全面深化改革，激发市场活力和社会创造力。

继续推进供给侧结构性改革。巩固扩大去产能成效，继续消化钢铁、水泥和电力等过剩产能，全面依法关停淘汰小散污落后产能、落后企业。深化去库存举措，加大自住购房支持力度，出台商业用房租售消化政策，力争棚改货币化安置比例达到100%。有效防风险、去杠杆，重点关注企业债兑付、贷款不良、政府债管控等工作，切实防范区域性金融风险。持续加大降成本力度，持续降低企业融资、用能、用水、物流等生产性成本，尽可能压减税费、审批等制度性成本，社会物流总费用占生产总值比重由16%降低至14%。实施补短板专项工程，围绕实现有效供给和有效需求动态平衡、增加公共产品和公共服务供给、完善社会民生兜底保障政策等方面，打出一套“组合拳”，同步补齐发展硬短板和软短板。

重点推进农村“三权分置”改革。基本完成农村土地承包经营确权登记颁证，扩大土地经营权抵押贷款试点范围，加强土地流转和规模经营管理服务，落实集体所有权，稳定农户承包权，放活土地经营权，充分释放土地“红利”。积极发展多种形式的适度规模经营，鼓励农户通过互换承包地、联耕联种等多种方式开展集中耕种，支持通过土地流转、土地托管、土地入股等多种形式发展适度规模经营。加快农村集体产权制度改革，探索建立城乡统一用地市场，稳妥推进农村土地征收、集体经营性建设用地入市、宅基地制度 “三块地”改革，让农村改革多点发力、多方获利。

积极推进投资领域改革。制定支持混合所有制经济发展的实施意见，为非公有制经济发展扫清障碍、松绑助力。支持民间资本参与国有企业改革，进入基础性公共项目领域。出台进一步促进民间投资健康发展的政策，使非公有制经济占经济总量比重达60%以上。

着力推进重要领域和关键性改革。深入推进“放管服”改革，制定投资核准事项清单、工商登记前置审批事项清单、企业设立后的经营许可清单、市场准入负面清单，推行工程建设项目多评合一、多审合一、多图连审、联合验收，以清单管理推动减权放权。组建成立行政审批服务局，积极推进相对集中行政许可权改革试点工作。大力推行“互联网+政务服务”，完成“政务服务与网上审批平台”建设，实现“一号申请、一窗受理、一网通办”。加快国企国资改革。稳妥推进财税体制改革。完成事业单位和国有企业公车改革。开展资源性产品价格改革，制定分质分价用水政策，扩大居民用电阶梯电量级差，形成有利于改善环境、节约资源、满足用户多层次需求的价格机制。

（九）全方位扩大开放，构建八面来风的开放新格局。充分发挥优势，构建引领呼包银榆、承接京津冀鲁、融入“一带一路”、八面来风的对外开放和区域联动发展新格局。

深入落实《建设中蒙俄经济走廊规划纲要》，全面加强电子口岸公共服务平台、国际快件通道、“无水港”建设，探索筹建中蒙、中俄经贸合作示范园区，推动由我市始发至欧洲的国际集装箱专列运行常态化，不断加强同俄蒙及欧亚其他地区的经贸、产业和人文交流合作，深度融入国际产业分工合作体系，提升对外开放水平。

积极参与京津冀协同发展。加快完善公路、铁路、航空等交通网建设规划，全力打造出区达海的进出通道。抓好产业园区、科技园区、物流园区共建，着力建设承接京津冀鲁产业转移的重要基地。

引领带动呼包银榆协同发展。认真落实《呼包鄂协同发展规划纲要（2016－2020年）》，进一步强化呼包银榆经济区合作机制，以和林格尔新区为载体，推动基础设施互联互通、产业发展互补互促、公共服务共建共享、要素配置对接对流，逐步形成以我市为中心的区域性合作发展格局，构建自治区向北开放中心区和国内协作大平台。

创新开放合作体制机制。用好国家西部大开发政策和国务院促进内蒙古经济社会又好又快发展的支持政策，建立健全我市招商合作政策体系，制定一批针对性强、有吸引力的差别化政策，强化政策执行和落实，提高招商引资效率。立足现有发展基础和自身特色，围绕推动大数据等战略性新兴产业发展，加快储备一批合作项目。大力发展跨境电子商务，积极争取批准国家互联网骨干网直联点、国家跨境电子商务综合试验区，加快形成以出口为主、进口为辅、线上线下有序结合的跨境电子商务发展格局。

（十）大力发展社会事业，提高基本公共服务水平。

坚持教育优先发展。新建续建公办中小学、幼儿园25所，深入推进考试招生制度改革。全面实施“农村学前教育推进工程”，制定乡村教师支持计划，提升农村教育质量。研究制定《学前教育第三期三年行动计划》，扩大普惠性学前教育供给。促进义务教育均衡发展，所有旗县区全部通过国家义务教育发展基本均衡县评估认定。加强教师队伍建设，下大力解决教师编制不足问题。加快职业教育基础能力建设，完善现代职业教育体系。制定加快发展民族教育实施方案，扶持和规范民办教育，关心支持特殊教育发展。

推动文化事业繁荣发展。以创建国家公共文化服务体系示范区为目标，重点建设乌兰夫纪念馆、儿童探索博物馆、内蒙古革命历史博物馆等一批文化场馆，积极申报国家级抗战遗址名录，加快推进“文化客厅”、市四区小剧场等项目建设，抓好县、乡、村三级文化馆、图书馆、公共文化综合服务中心等项目实施，提升公共文化服务供给能力。推进媒体融合和广播电视事业健康发展。精心办好第二十六届中国金鸡百花电影节、第十八届昭君文化节和第七届中国·呼和浩特少数民族文化旅游艺术活动，加大地方戏曲传承保护力度，创作一批优秀文艺作品，扩大地域文化、民族文化影响力。深化群众性精神文明创建活动，全力创建全国文明城市。

推进健康呼和浩特建设。优化医疗卫生资源布局，抓好自治区和市级重点医疗卫生项目建设，实施市第二医院迁建，在城市东部建设一所三级甲等心脑血管及儿童专科医院，在城市南部规划建设内蒙古蒙医药学院和一所三级甲等综合医院、内蒙古蒙医药研发中心及重点实验室。强化社区卫生服务中心和乡镇卫生院投入，做好乡村卫生室硬件配套和人员队伍建设，大力推广“市考县聘乡用”机制、农村医生“村来村去”制度，逐步解决基层医疗卫生服务力量不足问题。加强重大疾病预防控制，做好妇女儿童、老年人、残疾人、流动人口等人群健康服务工作。

抓好体育事业发展。以建设体育强市为目标，深入开展全民健身运动，加强竞技体育发展。统筹推进健身娱乐、体育用品、体育彩票、运动休闲、电动赛车、马术竞技等体育产业发展，打造特色体育品牌。全面完成少数民族群众文化体育运动中心、国家（北方）足球训练基地、游泳跳水馆、体育馆等重点项目，为首府市民提供更多健身运动场所。

深入实施《呼和浩特市妇女儿童发展纲要（2011—2020年）》。加强国防动员和后备力量建设，落实各项优抚安置政策，推动军民融合发展。统筹做好红十字、残疾人和慈善事业，发挥好工会、共青团、妇联等人民团体的桥梁纽带作用。全面贯彻党的宗教工作基本方针，发挥宗教界人士及信教群众的积极作用，扎实做好统计、档案、气象、地震、外事侨务等工作，推动各项社会事业再上新台阶。

（十一）织密扎牢民生保障网，增进各族人民福祉。落实以人民为中心的发展思想，从解决群众最关心最直接最现实的利益问题入手，做好普惠性、基础性、兜底性民生建设，坚决守住民生底线。

积极扩大就业创业。实施就业优先战略和更加积极的就业创业政策，鼓励多渠道、多形式就业创业。抓好高校毕业生等重点群体就业，落实就业促进和创业引领计划，鼓励高校毕业生到基层工作、开展创新创业。加强职业技能培训，有序组织农民工就近就地转移就业。全面落实就业援助政策，确保“零就业家庭”动态清零。积极做好破产转制企业职工安置工作，妥善解决去产能过程中的失业再就业问题，保障职工的合法权益。

持续提高城乡居民收入。深化收入分配制度改革，健全与经济增长相适应的工资增长机制，促进形成公正合理的收入分配格局。逐步提高最低工资标准，增加中低收入人群收入。继续提高退休人员基本养老金水平，健全企业职工最低工资调整机制，引导企业职工工资合理增长，使我市总体工资水平在全国省会城市中排名前移。多措并举拓宽农民增收渠道，持续稳定增加农民收入，不断缩小城乡之间、区域之间收入差距。

完善社会保障体系。实施全民参保计划，抓好跨省异地就医，加快推进社会保障“一卡通”进程。完善养老保险和医疗保险政策，建立全市统一的城乡居民基本医疗保险制度，将更多进城务工农民、失地农民、灵活就业人员纳入职工养老保险和城镇职工医疗保险。加大对困难群众的救助力度，统筹推进扶老、助残、救孤、优抚等福利事业发展。完善重大疾病保险机制，做好医疗救助工作，全额资助城乡低保、农村五保、城镇“三无”人员等困难群众参加城乡居民基本医疗保险，大幅提高旗县城镇低保、农村低保、五保供养和残疾人“两项补贴”标准。

构建和谐稳定屏障，提高“两个保障”水平。全面贯彻党的民族政策，深入开展民族团结进步教育和创建活动，巩固发展各民族守望相助、团结奋斗的良好局面。加强基层社区建设，加大社区活动场所、服务场所投入，健全完善社区综合服务功能，夯实基层基础。深入推进“平安首府”建设，加强以信息化为支撑的社会治安防控体系建设，全面推进“平安365”工程，严厉打击各类违法犯罪活动。全面开展社会矛盾大排查大化解、信访积案“百日攻坚”、以“忠诚干净担当”为主题的三个专项行动，维护首府良好的社会秩序。强化安全生产责任落实，开展重点领域专项整治，加强特种设备安全监管，坚决遏制重特大安全事故发生。强化食品药品安全监管，加快建立科学完善的食品药品安全治理体系，让广大群众饮食用药无安全之忧。

在此基础上，我们将努力办好一批与群众利益息息相关的民生实事，让改革发展成果更多更公平地惠及广大人民群众。

（十二）加强政府自身建设，努力打造人民满意政府。严格遵守党的政治纪律和政治规矩，进一步增强“四个意识”特别是核心意识、看齐意识，更加坚定自觉地在思想上政治上行动上同以习近平同志为核心的党中央保持高度一致，更加扎实地把党中央、国务院、自治区党委、政府和市委的各项决策部署落到实处。

建设法治政府。坚持依法行政，做到法定职责必须为、法无授权不可为，始终把政府工作纳入法治轨道。认真执行市人大及其常委会的决议、决定，主动向市人大及其常委会汇报工作，自觉接受市人大工作监督、法律监督。加强社会主义协商民主建设，接受市政协民主监督。充分听取人大代表、政协委员、民主党派、工商联、无党派人士和人民团体意见建议。积极推进智能人才库、法律专家库建设，提高依法决策、科学决策水平。加强法制教育宣传，深入推进“七五”普法。支持法院、检察院依法独立公正行使职权。

建设实干政府。树立“无为就是过、无为必问责”的理念，发扬肯干能干会干快干的实干精神，撸起袖子干工作、扑下身子谋发展，坚决纠正和问责懒政、怠政、不担责、不作为等行为。坚持问题导向，组织开展双向“问政”工作，真正把解决群众反映强烈的突出问题作为开展工作的出发点和落脚点。

建设诚信政府。全面推进政务公开，推行“双随机、一公开”制度，打造透明电子政务，维护公民知情权。发挥政府在诚实守信方面的表率作用，政府制定的各项优惠政策坚决执行，政府承诺的所有民生实事必须兑现，切实提高政府公信力。强化诚信教育和宣传，推动信用文化建设，营造公开、公平、公正的社会诚信氛围。

建设廉洁政府。严格履行全面从严治党主体责任，深入推进政府系统党风廉政建设。始终高举反腐利剑，不断完善重点领域和关键环节的制度设计，以零容忍态度惩治腐败。驰而不息推进作风建设，健全改进作风长效机制，巩固扩大“两学一做”学习教育成果。建立完善正向激励机制和容错纠错机制，旗帜鲜明地褒扬担当者、支持担当者、保护担当者，让广大干部愿意干事、敢于干事、能干成事。

各位代表，今年我们将迎来自治区成立70周年大庆，这是自治区政治生活中的一件大事。呼和浩特市作为活动“主会场”，高标准、高质量、高水平地做好迎庆各项工作，是我们必须履行的重大政治责任和历史使命。我们要精心谋划、扎实推进、坚决完成场馆建设、环境整治、氛围营造、交通管理、安全稳定等各项迎庆任务，确保70周年大庆各项活动顺利进行、圆满成功。

各位代表，新的一年，我们担负着新的艰巨发展任务、承载着新的重大历史使命。让我们更加紧密地团结在以习近平同志为核心的党中央周围，在自治区党委、政府和市委的坚强领导下，守望相助、团结奋斗，勇于担当、一往无前，为把祖国北疆这道风景线打造得更加亮丽而努力奋斗，以优异的成绩迎接党的十九大胜利召开，庆祝自治区成立70周年！
